# RUS6, a DUF647-containing protein, is essential for early embryonic development in *Arabidopsis thaliana*

**DOI:** 10.1186/s12870-021-03011-8

**Published:** 2021-05-25

**Authors:** Nathaniel Perry, Colin D. Leasure, Hongyun Tong, Elias M. Duarte, Zheng-Hui He

**Affiliations:** Department of Biology, San Francisco State University, CA 94132 San Francisco, USA

**Keywords:** RUS Gene Family, DUF647, Embryo Development, Complementation, Arabidopsis

## Abstract

**Background:**

The *Arabidopsis RUS* (*ROOT UV-B SENSITIVE*) gene family contains six members, each of which encodes a protein containing a DUF647 (domain of unknown function 647) that is commonly found in eukaryotes. Previous studies have demonstrated that RUS1 and RUS2 play critical roles in early seedling development. All six *RUS* genes are expressed throughout the plant, but little is known about the functional roles of *RUS3*, *RUS4*, *RUS5* and *RUS6*.

**Results:**

We used a reverse-genetic approach to identify knockout mutants for *RUS3*, *RUS4*, *RUS5* and *RUS6*. Each mutant was confirmed by direct DNA sequencing and genetic segregation analysis. No visible phenotypic differences were observed in *rus3*, *rus4*, or *rus5* knockout mutants under standard growth conditions, but *rus6* knockout mutants displayed a strong embryo-lethal phenotype. Two independent knockout lines for *RUS6* were characterized. The *rus6* mutations could only be maintained through a heterozygote, because *rus6* homozygous mutants did not survive. Closer examinations of homozygous *rus6* embryos from *rus6*/ + parent plants revealed that *RUS6* is required for early embryo development. Loss of *RUS6* resulted in embryo lethality, specifically at the mid-globular stage. The embryo-lethality phenotype was complemented by a *RUS6::RUS6-GFP* transgene, and GFP signal was detected throughout the embryo. Histological analyses with the *β-glucuronidase* reporter gene driven by the *RUS6* promoter showed tissue- and development-specific expression of *RUS6*, which was highest in floral tissues.

**Conclusion:**

Our data revealed that *RUS6* is essential for early embryo development in *Arabidopsis*, and that the *RUS* gene family functions in multiple stages of plant development.

**Supplementary Information:**

The online version contains supplementary material available at 10.1186/s12870-021-03011-8.

## Background

A wide variety of internal and external factors regulate and control plant development at various stages [1]. Many basal cellular functions are required for each specific developmental stage. A variety of approaches have been used to identify genes that are required for tissue or organ development, including embryonic, shoot, root, seedling, and flower development. For example, reverse genetics approaches have been successfully used to identify genes essential for embryonic development in *Arabidopsis* [1, 2]. As of 2020, 510 EMBRYO-DEFECTIVE (EMB) genes, which are required for successful embryo development, have so far been identified and described in *Arabidopsis* [3]. These genes are required for embryonic viability, with specialized functions ranging from synthesis of macromolecules (DNA, RNA and protein) to cellular structure and metabolism. It is likely that more *EMB* genes will be identified, and there are an estimated 750 to 1000 *EMB* genes in *Arabidopsis* [3–5]. One strategy to uncover additional *EMB* genes is to focus on gene families where loss of one or more members is known to cause developmental arrest at some stage.

The *ROOT UV-B SENSITIVE 1* (*RUS1*) gene, which encodes a protein that contains a DUF647 (DOMAIN OF UNKONWN FUNCTION 647), was first identified in *Arabidopsis* as an essential player in *Arabidopsis* early seedling development [6]. Knockout mutants for *RUS1* displayed an arrested phenotype following germination in a ultraviolet B (UV-B) influenced way [6]. Further studies identified a mutation in a homologous gene, *RUS2*, which showed identical phenotypes to those of the *rus1* knockout mutant [7]. Homozygous *rus1* and *rus2* single mutant, and *rus1 rus2* double mutant seedlings displayed an identical post-germination developmental arrest phenotype. This developmental arrest phenotype could be partially rescued by growing seedlings in MS media with high concentrations of vitamin B6, and/or reducing UV-B exposure to MS media plates with standard vitamin B6. Genetic suppressor studies revealed that specific mutations affecting the vitamin B6 binding pocket of ASPARTATE AMINOTRANSFERASE2 (ASP2) suppressed the *rus1* and *rus2* phenotype [8]*.* These findings suggest that RUS1 and RUS2 may interact with ASP2 to regulate early seedling development through vitamin B6 homeostasis [8].

Interestingly, the *RUS2* gene encodes another DUF647-containing protein, and both *RUS1* and *RUS2* share similar expression patterns [7]. Furthermore, RUS1 and RUS2 proteins were shown to physically interact in a DUF647-dependent manner, and the interaction appeared to be essential for their physiological function [7]. *RUS1* and *RUS2* were independently identified as *WXR3* (*WEAK AUXIN RESPONSE3*) and *WRX1*, respectively, when genetic mutants were screened for defects in auxin response and auxin-related growth defects [9, 10]. Both *wrx1* and *wrx3* mutants accumulated auxin in the hypocotyl and cotyledons, with reduced auxin levels in the root apex [9, 10]. These studies suggest that RUS1 and RUS2 may play important roles in physiological processes that include UV-B responses, vitamin B6 homeostasis, and polar auxin transport.

About 24% of all proteins annotated in the Pfam database are categorized as proteins containing a “domain of unknown function” (DUF). Of the 16,295 protein families in the Pfam database, 3,892 are DUF proteins, and the functional roles of these DUF proteins are yet to be experimentally characterized [11]. Taxonomically, DUF-containing proteins are widely distributed in both prokaryotes and eukaryotes. Studies suggested that many DUFs are likely biologically essential [12]. Proteins containing DUF647 (Pfam family PF04884) are widely distributed in eukaryotic species across both the plant and the animal kingdom [7]. Genetic and molecular studies with RUS1 and RUS2 suggested that DUF647 may serve as a protein–protein interacting domain, and the protein–protein interaction between RUS1 and RUS2 via DUF647 is required for *Arabidopsis* early seedling development [8]. In addition to *RUS1* and *RUS2*, the *Arabidopsis* genome contains four additional *RUS* genes named *RUS3*, *RUS4*, *RUS5* and *RUS6*, Little is known about the functional roles of these four *RUS* genes. Assignments of functions for DUF families often depends on making assumptions from the functions of the characterized members. It is currently unknown whether other DUF647-containing proteins are involved in any specific developmental processes. Here we present a comprehensive genetic characterization of the *RUS* gene family. Our results demonstrate that null mutations for *RUS6* result in complete disruption of *Arabidopsis* embryogenesis by the mid-globular stage. The severe embryonic lethality phenotype in *rus6* mutants, and *RUS6* expression in flowers, suggest that the *RUS* gene family plays diverse functional roles in multiple developmental stages from embryonic development to reproduction.

## Results

### The *RUS* gene family is found throughout eukaryotes and was expanded in algae

We previously reported that *ROOT UV-B SENSITIVE1* (*RUS1*) and *RUS2* are required for post-germination growth in *Arabidopsis*, and that they likely play a role in vitamin B6 (pyridoxal-5’-phophate) homeostasis. The *RUS1* and *RUS2* genes both encode proteins that contain a DOMAIN OF UNKNOWN FUNCTION 647 (DUF647) [6]. The *Arabidopsis* genome encodes for six DUF647-containing proteins (*RUS1* through *RUS6*). RUS proteins are found in most eukaryotic species, including all plants, and most fungus and animals. We previously identified *RUS3* as the clear ortholog to the single *RUS* gene found in most animal genomes [7]. All plant genomes analyzed were found to encode for multiple RUS proteins, usually six or more. Protein sequence analyses identified clear orthologs of *RUS1*, *RUS2*, *RUS3*, and *RUS6* in all plant genomes. The genomes of rice and the moss *Physcomitrella patens* each contained recent duplications of *RUS6*, but the rice genome lacked a clear RUS4 ortholog, and the *P. patens* genome lacked a clear *RUS5* ortholog. The genome of the gymnosperm *Pinus sylvestrus* contained orthologs for all six *RUS* genes (Figure S1). Interestingly, we also identified orthologs for all six *RUS* genes in the genome of a Charophyte algae, *Klebsormidium nitens*, which branched from the plant lineage at least 700 million years ago [13]. Therefore, the expansion of the *RUS* gene family into the current set of six genes occurred long before the evolution of the embryophytes began.

### Identification and analysis of knockout mutants for *RUS3*, *RUS4*, and *RUS5*

**Fig. 1:**
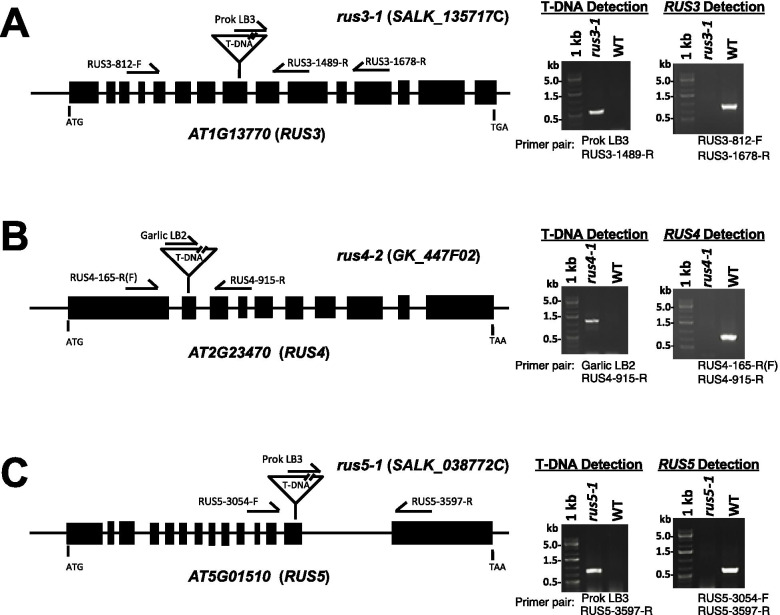
Identification and molecular characterization of T-DNA knockouts for *RUS3*, *RUS4* and *RUS5*. **a** T-DNA insertion position in the *RUS3* gene (*AT1G13770*) was detected and confirmed in the SALK_135717C T-DNA line. **b** T-DNA insertion position in the *RUS4* (*AT2G23470*) gene was detected and confirmed in GK-447F02 T-DNA line. **c** T-DNA insertion position in the *RUS5* gene (*AT5G01510*) was detected and confirmed in SALK_038772 T-DNA line. Filled boxes indicate exons. Positions for the start (ATG) and stop codons (TGA in *RUS3*; TAA in *RUS4* and *RUS5*) are indicated. Positions of the primer pairs used to detect either the presence of the T-DNA insertions or amplify the wild-type genomic sequence are indicated. Gel images of DNA fragments amplified with pairs of primers as indicated (Primer pair) below the gel image, from either the corresponding T-DNA line or the WT (wild type) are shown at the right side. Sizes of DNA ladder (1 kb) bands are indicated in kb (kilobase)

In an effort to further understand the functional roles for all *RUS* members, we screened and identified knockout mutants for *RUS3* (*AT1G13770*), *RUS4* (*AT2G23470*), *RUS5* (*AT5G01510*) and *RUS6* (*AT5G49820*). Potential T-DNA insertional lines were identified in the public database and verified by gene-specific PCR markers. Homozygous knockout mutants were identified for *RUS3* (two lines: *SALK_135717C* and *SALK_042033C,* which are *rus3-1* and *rus3-2,* respectively)*, RUS4* (one line: *GK-447F02-024,530*) and *RUS5* (one line: *SALK_038772C*) (Fig. 1). All mutant lines contain T-DNA insertions in exons (Fig. 1) (Figure S2). Homozygous mutants for all three genes (*RUS3*, *RUS4*, and *RUS5*) were isolated, suggesting that mutations in these three genes do not cause embryo lethality (Fig. 1). No noticeable morphological differences were observed between these mutants and the WT (Col-0) plants when grown under standard growth conditions.

### Loss of function in *RUS6* (AT5G49820) results in embryo lethality

**Fig. 2:**
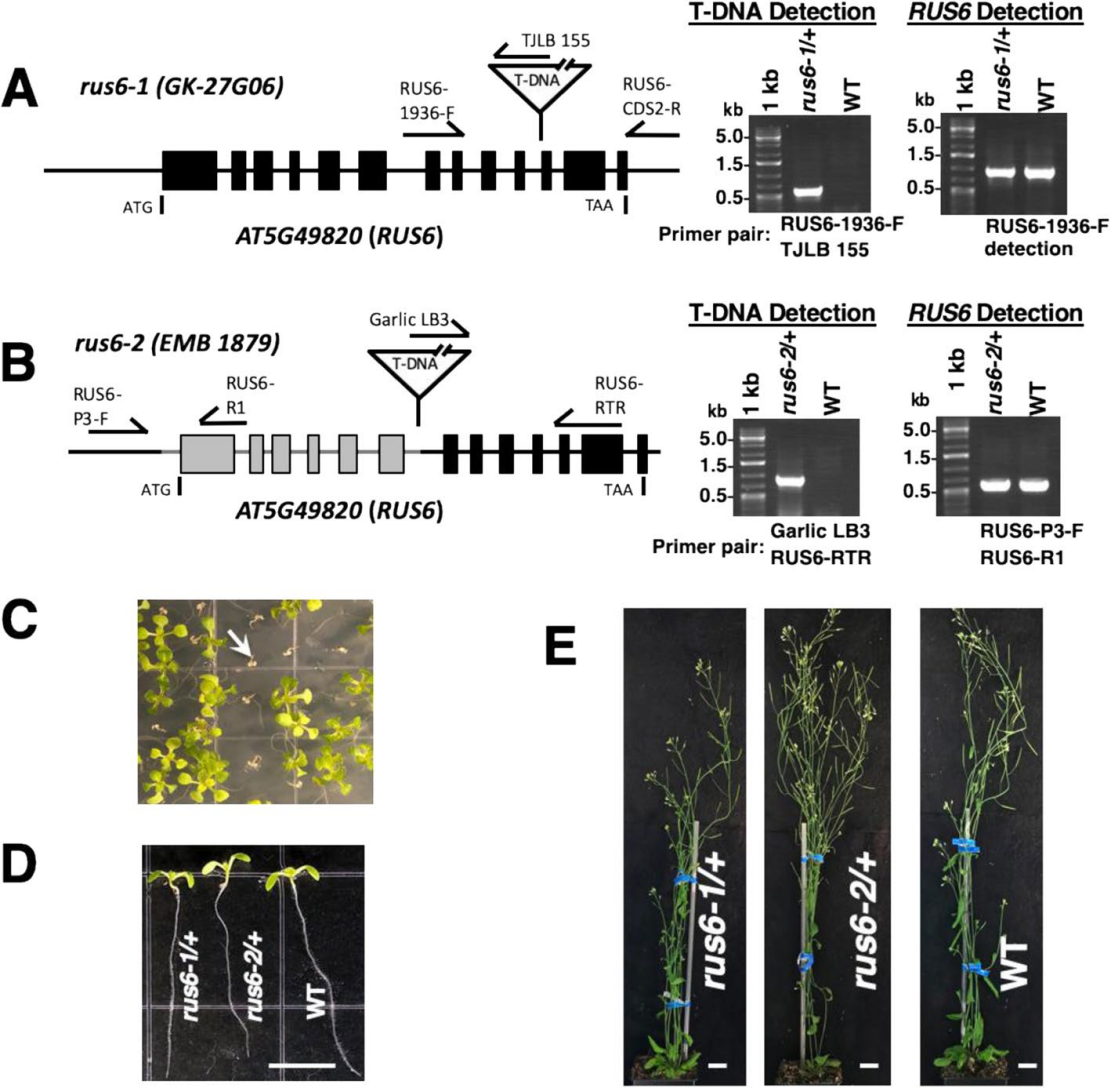
Identification and molecular characterization of heterozygous T-DNA lines with *RUS6* gene interruptions. **a** and **b** T-DNA insertions in *RUS6* gene (*AT5G49820*) were detected in two T-DNA lines: *GK027G06* and *emb1879*. Filled boxes indicate *RUS6* exons, greyed-out portions indicate deletions. Positions for the start codon (ATG) and the stop codon (TAA) of *RUS6* are indicated. Locations of T-DNA insertion in the *RUS6* gene and PCR primers used to detect either the T-DNA insertion (RUS6-1936F and TJLB155 for *GK-27G06* line; Garlic LB3 and RUS6-RTR for *emb1879* line) or a wild-type *RUS6* fragment (RUS6-1936F and RUS6-CDS2-R for the *GK*-*027G06* line; RUS6-P3-F and RUS6-R1 for the *emb1879* line) are indicated. Gel images of PCR detection of either the T-DNA (T-DNA Detection) or the wild-type *RUS6* fragments (*RUS6* Detection) are shown (right panels). For both *GK-27G06* and *emb1879*, *rus6* mutant alleles (*rus6-1* and *rus6-2*) can only be maintained as a heterozygote (*rus6*/ +). 1 kb: 1 kb marker. kb: kilobase. **c** Segregation of sulfadiazine-resistance marker in offspring of self-fertilized *rus6-1*/ + . 10-days-old seedlings were grown on MS plates containing sulfadiazine. Healthy green seedlings are individuals carrying the sulfadiazine-resistance maker and unhealthy brown seedlings (indicated by an arrow) are WT seedlings. **d** and **e** Heterozygous mutants (*rus6-1*/ + and *rus6-2*/ +) are indistinguishable from the wild-type (WT) at various stages of development. **d** Representative images of 7-days-old seedlings (*rus6-1*/ + , *rus6-2*/ + , WT) grown on MS plates are shown (bar = 0.5 cm). **e** Representative mature plants with inflorescences (42-days-old) are shown (bar = 2 cm)

Two T-DNA insertion lines (*GK278G06* and *emb1879/cs16037*) were identified for *RUS6* (*AT5G49820*), and verified by PCR markers and direct sequencing (Fig. 2a, b; Figure S2). *GK278G06* was obtained from Gabi-Kat [14] (https://www.gabi-kat.de/) and confirmed to have a *pAC106/pAC116* T-DNA insertion in exon 11 (Fig. 2a). The *cs16037/EMB1879* line was obtained from ABRC (Arabidopsis Biological Resource Center). The *cs16037/EMB1879* line has a deletion from the promoter region until intron 6, which was replaced by the *pCSA104* T-DNA insertion (Fig. 2b). The deletion/insertion was verified by PCR markers and DNA sequencing (Figure S2). After confirming the mutations, we named *GK278G06* and *cs16037/emb1879* as *rus6-1* and *rus6-2*, respectively (Fig. 2).

We were unable to identify any homozygous *rus6* mutants in either of the initial seed stocks for *rus6-1* or *rus6-2*. In order to produce homozygous *rus6* plants, *rus6-1*/ + and *rus6-2*/ + were each self-fertilized and their progenies were grown. PCR-based genotyping was used to genotype individual progeny, but no homozygous *rus6* mutants were identified in the offspring of either heterozygous parent (*rus6-1*/ + , *n* = 121; *rus6-2*/ + *, n* = *14*), suggesting that homozygous *rus6* mutants are embryo lethal.

The *rus6-1* T-DNA insertion contains a sulfadiazine (Sul) resistance gene. We grew the offspring of self-fertilized *rus6-1*/ + plants in the presence of Sul, and observed that 65.27% of the seedlings displayed Sul resistance and 34.73% displayed Sul sensitivity (*n* = 262) (Table 1; Fig. 2c). These numbers were consistent with a 2:1 ratio of *rus6-1*/ + to + / + plants, which is expected if the homozygous *rus6-1* plants are absent. A subset (*n* = 17) of the Sul-resistant plants were PCR genotyped and were all identified as *rus6-1*/ + ; no homozygous *rus6-1* plants were found.

**Table 1:** Segregation of T-DNA insertion in two *rus6* knockout lines

Genotype (line)	Resistance type	Number resistant	Number sensitive	3:1 Segregation analysis		2:1 Segregation analysis	
				**%resistant / ** **%expected**	***p* value**	**%resistant / ** **%expected**	***p* value**
*rus6-1/* + *(GK-278G06)*	sulfadiazine	171	91	65.3% / 75.0%	< 0.001	65.3% / 66.7%	0.63
*rus6-2/* + *emb1879*	basta	141	72	66.2% / 75.0%	0.003	66.2% / 66.7%	0.88

The *rus6-2* T-DNA insertion confers Basta (glufosinate) resistance. In agreement with the *rus6-1* results, we again observed results consistent with a lack of homozygous *rus6-2* plants. 66.20% of samples displayed basta resistance, and 33.80% displayed basta lethality (*n* = 213) (Table 1). The basta resistant plants (*n* = 22) that were PCR genotyped were all *rus6-2/* + .

The Sul and Basta resistance results conformed to the expected 66.7% to 33.3% (2:1) segregation ratio for heterozygous to WT seedlings if homozygous progeny were missing (Table 1). Additionally, seed germination rates were comparable between the mutant lines and wild-type controls, suggesting that the seeds of homozygous *rus6* embryos were not produced. Thus, we hypothesized that the lack of *rus6* homozygotes was caused by early embryo lethality leading to seed abortion, rather than failed germination. Taken together, these results suggested that homozygous mutations in *rus6* result in embryo lethality.

### Loss of function in *RUS6* disrupts embryo development, leading to a white developing seed phenotype

**Fig. 3:**
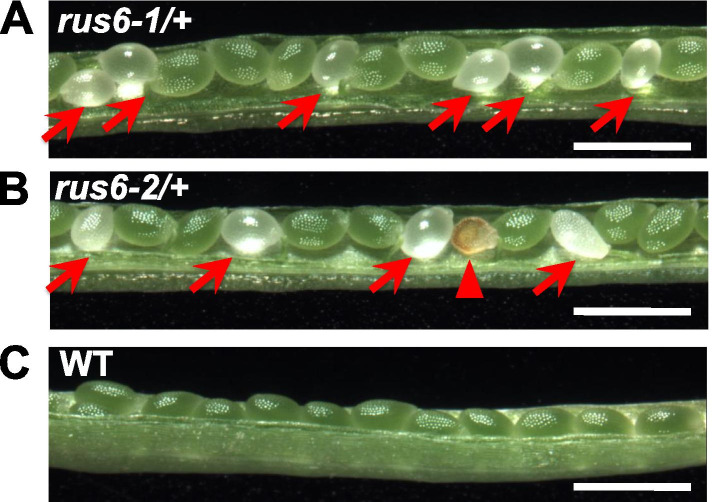
Embryonic lethality in heterozygous *rus6*/ + plants. **a** and **b** Developing siliques with embryos around mid-bent-cotyledon stage to late-bent-cotyledon stage from a *rus6-1*/ + plant **(a)** and a *rus6-2*/ + plant **(b)** were examined for seed development. **c** Siliques from WT were used as a control. Normal developing seeds are green and round. White (arrow) and brown (triangle) seeds, indicative of embryo lethality, are present in the siliques of *rus6-1*/ + and *rus6-2*/ + plants. Bar = 1 mm

We observed *rus6/* + mutant plants from germination through maturity, and found that all vegetative parts of the plant were indistinguishable from WT (Fig. 2d, e). To investigate the lack of homozygous *rus6* seeds, we opened *rus6*/ + siliques and characterized the developing seeds inside. While most of the developing seeds were green, similar to wild-type plants, we also observed a high percentage of developing seeds that were white, or brown and wrinkled, depending on the age of the silique (Fig. 3). We suspected that the white developing seeds contained the *rus6* homozygotes, and predicted that they represented 25% of the seeds in the silique, to fit a 3:1 ratio of green to white seeds [15]. A more extensive phenotypic analysis of developing seeds found 25.59% white or brown seeds in the siliques of *rus6-1/* + plants (*n* = 895) and 23.88% white or brown seeds in the siliques of *rus6-2*/ + plants (*n* = 356) (Table 2). These results suggested that *rus6* homozygotes are embryo lethal, and are the cause of the white developing seed phenotype.

**Table 2:** Analysis of aborted seeds in the two *rus6 T-DNA lines*

Genotype (line)	Green (normal)	Brown (aborted)	White (aborted)	% of aborted / % expected	*p* value
*rus6-1/* + *(GK-278G06)*	666	30	199	25.6% / 25.0%	0.69
*rus6-2/* + *EMB 1879*	271	14	71	23.9% / 25.0%	0.62
WT	460	7	0	1.50% / ~ 1	< < 0.01

### Complementation abolishes the *rus6* embryo lethal phenotype

Our initial analyses of the *rus6-1* and *rus6-2* mutations strongly suggested that at least one functioning copy of *RUS6* is required in *Arabidopsis* plants. To reduce the possibility of an additional T-DNA insertion somewhere in genome being fully or partially responsible for the *rus6* phenotype, we twice backcrossed *rus6-1*/ + plants to wild-type Col-0. The *rus6* phenotype remained consistent in the purified backcrossed line, which led strong support to the *rus6-1* mutation being the cause of the phenotype.

**Fig. 4:**
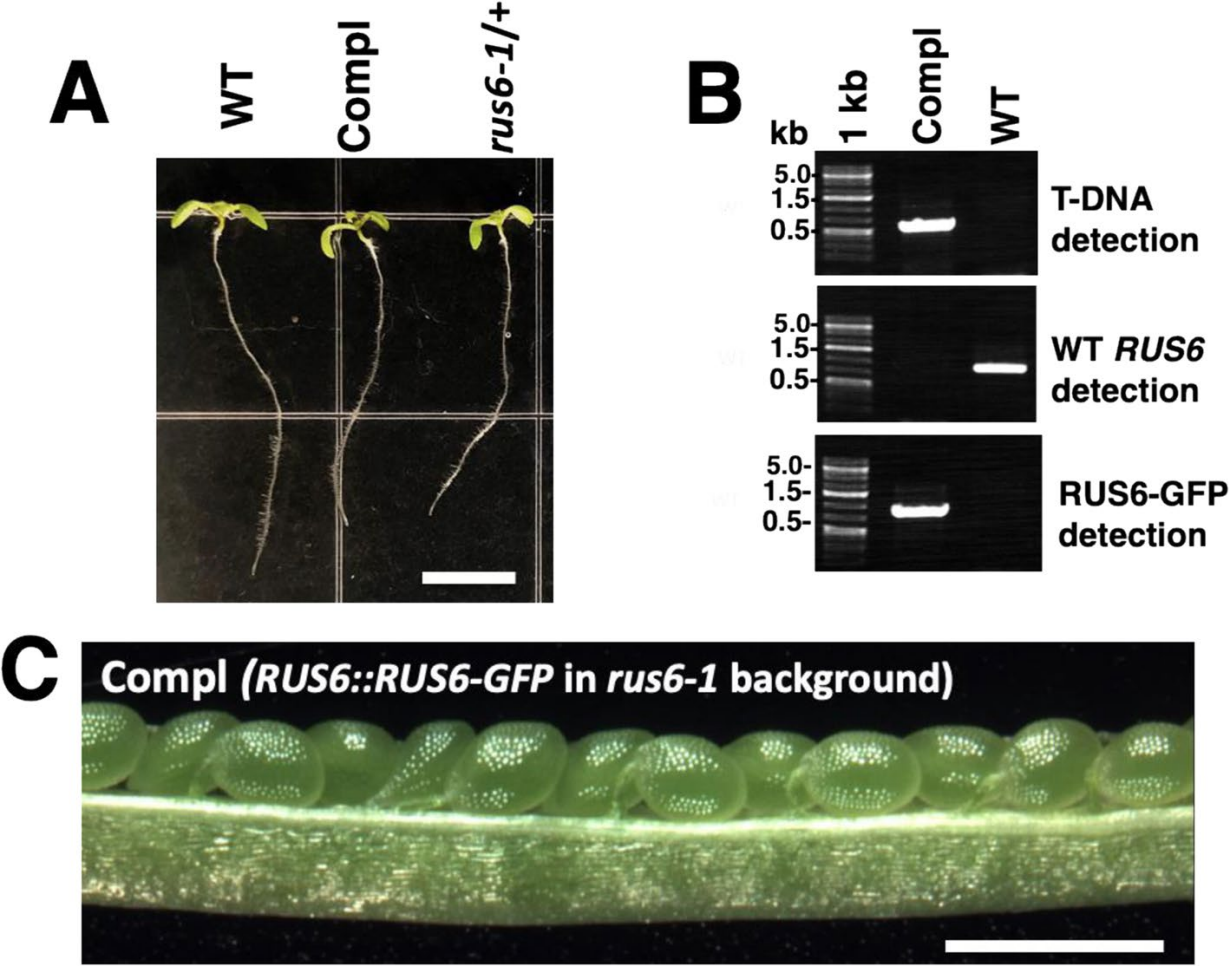
Complementation of homozygous *rus6-1* mutants. *rus6-1*/ + plants were transformed with a *RUS6::RUS6-GFP* construct, and homozygous *rus6-1* plants carrying *RUS6::RUS6-GFP* (Compl) were recovered. **a** Images of 7-days-old seedlings of WT (WT), the complementation line (Compl) (in homozygous *rus6-1* background), and the *rus6-1*/ + line, are shown. Bar = 0.5 cm. **b** The complementation line carrying *RUS6::RUS6-GFP* is *rus6-1* homozygous. Gel images of specific markers for T-DNA detection (T-DNA detection), WT *RUS6* (WT *RUS6* detection), and the *RUS6::RUS6-GFP* transgene (RUS6-GFP detection) are shown. 1 kb: 1 kb DNA ladder. kb: kilobase. **c** *RUS6::RUS6-GFP* rescues the *rus6-1* white seed phenotype. Image of a representative dissected silique from the *RUS6::RUS6-GFP* complemented line (in *rus6-1* homozygous background) is shown. Bar = 1 mm

In order to genetically complement the *rus6-1* mutation, we created a chimeric *pZP222* construct containing *RUS6-GFP* driven by the native *RUS6* promoter (*RUS6::RUS6-GFP*). The GFP tag was included for later analysis with fluorescence microscopy. *rus6-1*/ + plants were transformed using *Agrobacterium tumefaciens*, and T1 seeds were harvested and plated on antibiotic selection MS media. Two resistant T1 plants were identified and PCR analysis confirmed that they contained the *RUS6::RUS6-GFP* transgene. T2 seeds were collected from each line, and antibiotic selection and PCR genotyping was performed. We identified *rus6-1* homozygous plants in the T2, which contained at least one copy of the *RUS6:RUS6-GFP* transgene (Fig. 4a, b). The complementation of the *rus6* lethality phenotype by *RUS6:RUS6-GFP* demonstrated that the *rus6-1* mutation was responsible for the *rus6* mutant phenotype (Fig. 4c).

### *rus6* homozygous mutations prevent embryo development past the globular phase

To examine differences in embryo development between the white and green seeds in *rus6-1*/ + siliques, we performed Differential Interference Contrast microscopy (DIC) on developing seeds. Seeds from the same *rus6-1/* + silique were removed, cleared, and examined together, and the results were consistent across siliques analyzed. We initially performed microscopy on seeds from late stage siliques of *rus6-1*/ + plants, and observed that the white seeds completely lacked a detectable embryo. We then examined seeds from siliques of decreasing maturity, which resulted in an increase in the number of white seeds that contained embryos, which were never observed to be past the globular stage. Finally, we observed that in very young siliques all of the white seeds contained globular phase or earlier embryos. These results suggested that the *rus6* embryos were in fact initiated, but degraded and became undetectable after failing to advance past the globular phase.

**Fig. 5:**
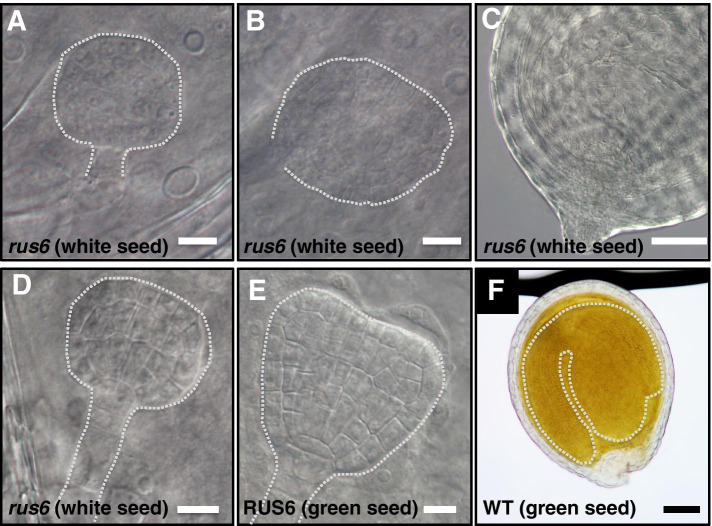
RUS6 is essential for normal embryonic development. Embryos dissected out from seeds of both *rus6-1*/ + and WT plants were examined by differential interference contrast (DIC) microscopy. Shapes of the observed embryo are traced with dotted lines to outline the positions of the embryos. Images of embryos from the same *rus6-1*/ + silique demonstrate that embryos from white seeds are severely developmentally delayed (~ 10 × smaller) as compared to those from green seeds, and fail to develop past the mid- to late-globular phase. **a-b** Defective embryos from white seeds stalled at the mid- to late-globular phase show a distorted/absent suspensor, a lack of identifiable columella, and initial stages of deterioration. **c** A white seed from a more mature *rus6-1*/ + silique has no detectable embryo. **d** An arrested embryo from a white seed in mid-globular phase that has not yet begun to deteriorate. **e** A representative transition to early heart stage embryo from a WT silique. No *rus6-1*/ + white seed embryos were observed at this phase. **f** A representative late-bent-cotyledon stage embryo from a green seed shows normal development as compared to white seeds in the same *rus6-1*/ + silique shown in **a**, **b**, and **d**. Bar = 5 microns (**a, b, d, and e**). Bar = 50 microns (**c and f**)

The *rus6* embryos in white seeds were severely delayed, and unable to develop past the globular phase (Fig. 5a, b, c, and d). In contrast, the embryos inside developing green seeds, which were either *rus6/* + or wild-type, had normal developmental morphology (Fig. 5e and f). Additionally, the embryos in green seeds in each silique examined were all at a similar stage of development. The *rus6* embryos displayed altered morphology, and careful observations determined that they were unable to reach the transition phase. Images shown in 5A, 5B, 5D, and 5F were all taken from embryos in the same *rus6-1*/ + silique. The hypophysis or columella cells were either absent or distorted in such a way as to appear as part of the lower tier (Fig. 5b). Additionally, the suspensors of mutant embryos at this stage were difficult to detect and often appeared to be absent. Figure 5d shows an arrested embryo that has not yet begun to deteriorate. Once an embryo starts to deteriorate, it becomes difficult to be imaged for its internal details. Our analyses suggest that *rus6* mutant embryo development stalled at the mid- to late- globular phase, and that the embryos subsequently deteriorated leading to failed seed development.

### *RUS6* is expressed in the embryo

**Fig. 6:**
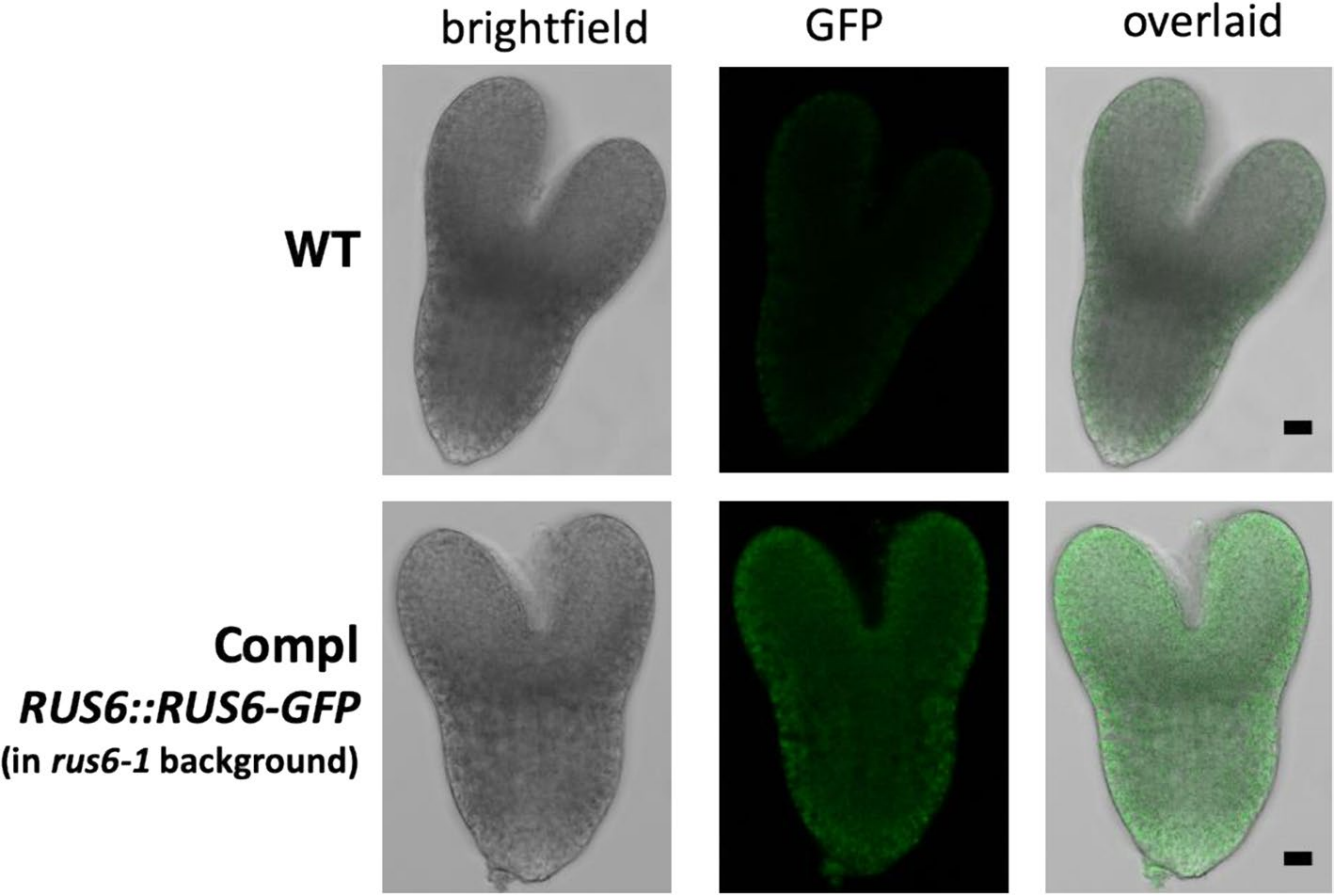
*RUS6* expression in developing embryo. Embryos from a *RUS6::RUS6-GFP* transgenic line (in homozygous *rus6-1* background) were dissected and examined under a Confocal microscope. An embryo from WT (WT) was used to as a control. A representative *RUS6::RUS6-GFP* embryo (Compl) is shown. Brightfield, GFP fluorescence, and overlaid images are shown as indicated. Bar = 5 microns

To observe *RUS6* expression in vivo, we analyzed GFP fluorescence in homozygous *rus6* mutants complemented by our *RUS6::RUS6-GFP* construct. To minimize the auto-florescence that comes with more developed tissues, we performed laser scanning confocal microscopy on embryos in the late heart to early torpedo stages. We detected GFP fluorescence in complemented embryos that was significantly above the background auto-fluorescence seen in the wild-type control (Fig. 6). Observation at higher magnifications revealed that RUS6 is not specifically localized to the cell wall, nucleus, mitochondria, or any diffuse organelle (Fig. S3). In contrast, fluorescence patterns suggested that RUS6 was mainly localized to some distinct round and punctate structures outside of the vacuole. The TargetP 1.1 predicted that RUS6 localizes to either the chloroplast or other cellular location [16].

### *RUS6* expression in vegetative and reproductive organs

In order to further evaluate *RUS6* expression patterns, we use a *pBI101* construct to generate a *RUS6::GUS* reporter gene. The *RUS6* promoter used in this reporter was the same region that was successfully used in the complementation of the *rus6-1* mutation. Following *Agrobacteria-*mediated transformation, selection and PCR analysis confirmed twelve primary (T1) transformants. We performed preliminary GUS staining on all twelve lines, and selected the two with the highest GUS expression levels for further imaging and analyses. T2 plants from line 12 yielded the highest GUS activity in the flowers, while T2 plants from line 1 had the highest expression for all other tissues. *RUS6::GUS* expression was observed to be subtle, surprisingly dynamic, and was only detected at specific stages of development.

**Fig. 7:**
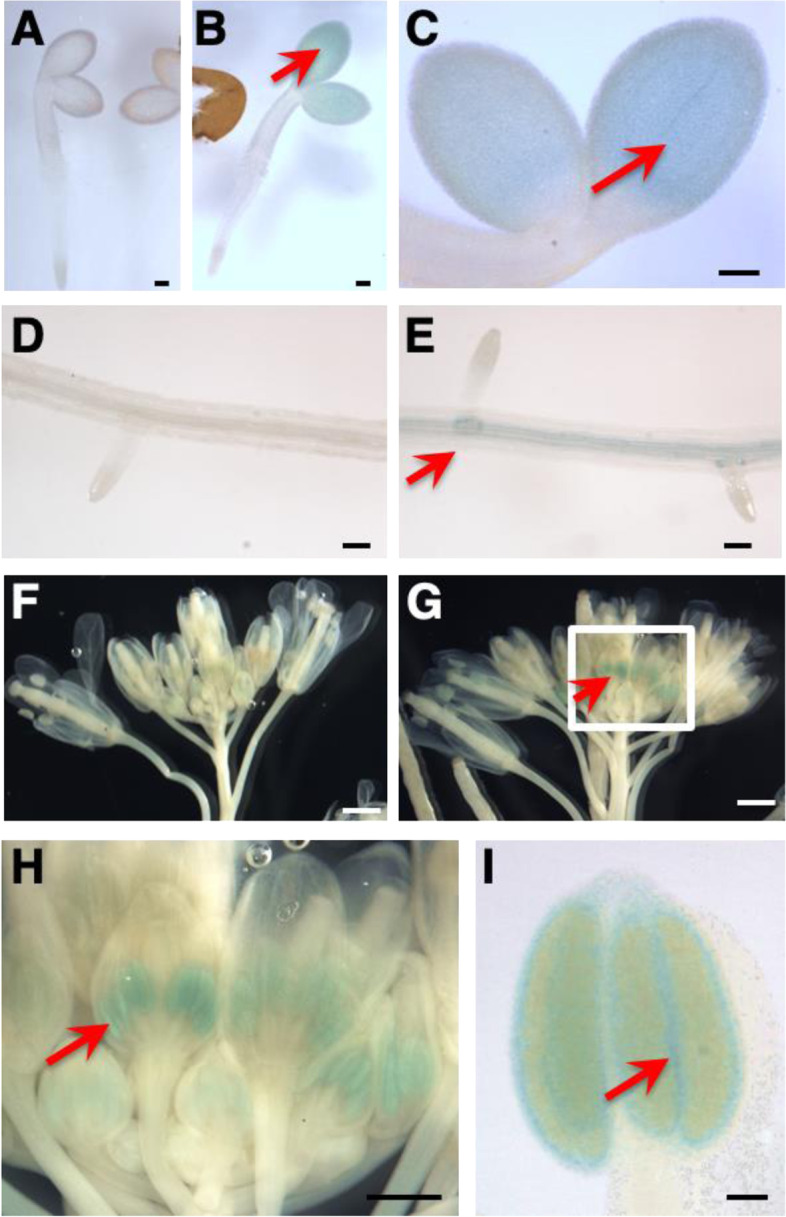
Organ- and tissue-specific expression of RUS6 expression.** b, c, e, g, h** and **i**, Images of histochemical staining of *RUS6::GUS* transgenic plants at various stages of development are shown. Plant organs or whole seedlings were incubated with 5-bromo-4-chloro-3-indolyl beta-D-glucuronide (X-Gluc) to detect β-glucuronidase activity (GUS), and then cleared before imaged. Locations of GUS staining are indicated by arrows. **a** WT seedlings are shown (bar = 0.1 mm). **b** and **c** GUS staining in 2-days-old transgenic seedlings (bar = 0.1 mm). **d** GUS staining of WT roots as a negative control (bar = 0.1 mm). **e** GUS expression in roots (20-days-old seedling) (bar = 0.1 mm). **f** WT flower negative control (bar = 1 mm). **g** GUS expression in flowers (bar = 1 mm). **h** Inset of the boxed area in (**g**) (bar = 0.5 mm). **i** GUS staining in anther (bar = 0.5 mm)

We stained one- through six-days-old *RUS6::GUS* light-grown seedlings grown vertically on M.S. plates. No GUS activity was observed in one day old seedlings, but two days old seedlings showed some degree of GUS activity in the cotyledons (Fig. 7a, b, c). We were unable to detect GUS activity in three- to six-days-old seedlings. Moreover, the GUS activity in two-days-old seedlings was only present in approximately 50% of the seedlings. This suggests that *RUS6* expression was dynamic and temporally specific to a precise stage of development.

In 20-days-old seedlings, GUS activity became clearly defined to the edges of the developing root primordia (Fig. 7e). GUS activity was also observed at this time in some lateral roots (Fig. 7d, e), and very faintly at mid-length in the primary root. Interestingly, some lateral roots, root tips, and root junctions showed GUS activity, while others did not. GUS expression did not appear to be based on the length of the lateral root, or any other observable marker of development.

GUS activity was not detected in leaves at any stage of development. However, flowers had the highest detected GUS activity in the plant, which was especially high in the anther (Fig. 7f, g, h, i). GUS activity was uniformly highest in the flower at stage 11, (as defined by Smyth et al., [17]). However, some flowers at later stages showed GUS activity, while others at the same stage of development did not. This pattern persisted even in flowers attached to the same inflorescence stem. Further investigation of dissected anthers revealed that GUS activity was especially high in the tapetum (Fig. 7i).

## Discussion

We have systematically identified and characterized *rus3*, *rus4*, *rus5*, and *rus6* knock-out mutants in *Arabidopsis*. Our study uncovered an essential role of *RUS6*, which encodes a DUF647-containing protein, in *Arabidopsis* embryo development. The *Arabidopsis* genome contains six genes that encode DUF647-containing proteins [7]. Two of the six genes, *RUS1* and *RUS2*, were previously characterized and are known to play critical roles in early seedling development. RUS1 and RUS2 work as functional partners to ensure the heterotrophic *Arabidopsis* embryo develops into an autotrophic seedling. Both *RUS1* and *RUS2* were also independently identified in a weak-auxin response genetic screen, suggesting that *RUS1* and *RUS2* regulate plant development by directly or indirectly affecting auxin distributions [9, 10]. Recently, a knockdown study of *RUS4* via artificial microRNA (amiR) suggested that *RUS4* plays a role in *Arabidopsis* reproductive development [18]. While homozygous *rus4* knockout mutants showed no obvious phenotype in their study and ours, down-regulation of *RUS4* mRNA by the amiR approach disrupted anther dehiscence, likely through the down-regulation of genes such as *NST1* and *NST2,* which are known to play roles in secondary cell wall thickening in the *Arabidopsis* anther endothecium [18]. In this study, our detailed genetic and molecular characterization of two knockout mutants for *RUS6*, *rus6-1* and *rus6-2*, clearly identified an indispensable role of *RUS6* in *Arabidopsis* embryonic development.

Embryonic lethality in *rus6* knockout mutants was confirmed by several lines of experimental evidence in our study. The *rus6-2* allele was identified earlier as an *emb* (*embryo-defective*) line with the name of *EMB1879* [2], and was tagged as *emb* by Syngenta (line CS16037, which contains a T-DNA insertion in the promoter region). However, the precise effect of this T-DNA insertion on the gene *AT5G49820* (*RUS6*) was not clear. A further report of embryo-lethality was later removed from the Seed Genes database (seedgenes.org), and recently *emb1879*/*AT5G49820* was excluded from a comprehensive review providing an updated dataset of 510 *EMB* genes [3]. Our analysis in this study provided convincing evidence that *emb1979* is in fact an embryo lethal, and should be included in *EMB* databases. We characterized the *emb1879/rus6-2* line along with another available T-DNA line (*rus6-1*), *GK-27G06*. No homozygotes could be obtained from either line, and defective seed development was observed in the progeny of heterozygotes for either mutation. These phenotypes are indicative of embryo lethality [19]. In a segregating population, the *rus6-1* allele and the *rus6-2* allele can be maintained only in individuals that are heterozygous for the mutant allele. Since each allele carries a T-DNA insertion with a specific selectable marker, the presence of the insertion was analyzed on growth media containing the selection reagent, either sulfadiazine or basta, respectively. For both alleles, the segregating populations showed that two thirds of the progeny were heterozygous for either the *rus6-1* or the *rus6-2* allele, and the remaining one third of the progeny were WT (Table 1). The quantitative data consistently suggested that homozygous *rus6* knockout mutants are embryo lethal.

Our study directly detected the nature of the *rus6* embryonic lethality through DIC microscopy. While a normal and developed embryo is green, an abnormal and aborted seed is white or brown [20]. Our quantitative analyses of the embryos in the developing siliques of plants heterozygous for either *rus6-1* or *rus6-2* confirmed that about 25% of the embryos were white or brown (Table 2). The ratios of the aborted embryos to the normal embryos further support the conclusion that *rus6* knockout results in embryonic lethality. In addition, further DIC microscopic examinations of the dissected white seeds demonstrated that embryo development appeared to arrest at stages prior to the transition stage. The aborted embryos showed various abnormalities, including distorted suspensors, or a lack of columella and/or suspensor. In some cases, no visible embryos were observed. It is highly likely that RUS6 functions at the early stages of embryonic development. As the globular stage was the latest stage detected in the *rus6* embryos, RUS6 appears to be required for the embryo to pass through to the transition stage. *RUS6* is known to be expressed in various tissues and at various developmental stages including embryo development [21, 22]. Proteomic analysis suggested that the RUS6 protein (Uniprot # Q93YU2) is ubiquitously expressed in all tissues analyzed (https://www.proteomicsdb.org/proteomicsdb/), but the highest RUS6 protein expression was found in mature embryos and pollen [21, 22]. Our RUS6-GFP Confocal data further demonstrated that RUS6 is ubiquitously expressed in late-torpedo stage embryo (Fig. S3). Although high levels of RUS6 expressions are found in pollen, no phenotypes related to gametophyte development were observed in rus6 knockout mutants. Our genetic data suggested that *rus6* gametophytes appeared to be functional and could complete fertilization at a normal rate. However, the embryos that were homozygous for either *rus6-1* or *rus6-2* failed to develop beyond the globular stage to form seeds. *RUS6* transcript level is also abundant during seed germination and early seedling development [23]. Whether and how RUS6 functions in early seedling development is yet to be analyzed. We complemented the homozygous *rus6* mutant with a construct containing the *RUS6* native promoter (*RUS6::RUS6-GFP*). To further understand the role of RUS6 in other developmental stages, a transgenic line in the homozygous *rus6* background carrying *RUS6-GFP* driven by an inducible promoter can be created in the future.

Although we have clearly established the requirement of *RUS6* in *Arabidopsis* early embryo development, how *RUS6* functions during this stage is currently unclear. A number of genes which have been documented as *emb* (*embryo-defective*), are known to be required for embryo development. Their protein products often perform essential cellular functions, and any major interference with these proteins could result in embryonic lethality [3]. RUS6 is predicted to be localized in the chloroplast [24]. Normal chloroplast functions are essential to embryonic development, and studies have shown that disruptions of a number of proteins that are essential to chloroplast functions result in embryo lethality [19]. In addition, many studies have established that auxin plays a role in embryo development [25], and specific spatiotemporal distributions of auxin are well documented [25]. These distribution patterns are achieved via local auxin biosynthesis by YUCCA members or by auxin transporters (efflux or influx transporters). RUS1 and RUS2 are known to have a strong connection with auxin distributions, as both *rus1* and *rus2* mutants showed altered auxin distributions. *RUS1* and *RUS2* are known to play a role in PLP (pyridoxal 5-phosphate, the bioactive form of vitamin B6) homeostasis [8] and PLP homeostasis plays an important role in regulating auxin homeostasis during postembryonic root development in *Arabidopsis* [26]. Furthermore, previous studies suggest that RUS1 and RUS2 may physically interact with PLP-binding proteins, such as aspartate aminotransferases, to regulate vitamin B6 homeostasis. Specific mutations to the PLP-binding pocket of the ASPARTATEAMINOTRANSFERASE (ASP) proteins suppress the *rus1* and *rus2* phenotypes.

Our study completed the identification of T-DNA knockout mutant for all six members of the RUS gene family. No observable phenotype was detected in homozygous mutants for *rus3*, *rus4*, and *rus5*, suggesting genetic redundancy for those three *RUS* genes. We also created double mutants for different gene pairs (*rus3 rus4*, *rus3 rus5*) and no observable phenotypes were found in these double mutants (Fig. S10). Homozygous triple mutants for the three genes could be created to test if these three members indeed share functional redundancy, although the embryo lethality of *rus6* presents obvious limitations with genetic crosses, and would require a transgenic line in the homozygous *rus6* background carrying *RUS6* driven by an inducible promoter. Future studies on various combinations of *rus3, rus4*, *rus5,* and *rus6* mutants under various physiological conditions will help determine the genetic roles of these *RUS* members. *RUS* genes are widespread in multicellular organisms and our previous phylogenetic analysis of the *RUS* genes suggested that *RUS3* is the ortholog to the *RUS* genes found in animals [7]. It is therefore surprising that no observable phenotypes were detected in *rus3* knockout mutants in *Arabidopsis* under normal growth condition. Species in the animal kingdom have a single *RUS* gene in each genome, whereas species in the plant kingdom have multiple (5–16) *RUS* genes. How *RUS* genes function in other multicellular organisms is largely unknown. Our current study with four of the *Arabidopsis RUS* genes provides experimental evidences that can help guide future efforts to discover how the *RUS* family functions in *Arabidopsis* and other species.

## Conclusion

The Arabidopsis genome contains six *RUS* genes, which all encode DUF647-containing proteins. Two of these genes, *RUS1* and *RUS2*, have roles in early seedling development, vitamin b6 homeostasis, and auxin transport. In this paper we analyzed knock-out mutants for the remaining *RUS* genes (*RUS3* through *RUS6*). Interestingly, homozygous *rus3*, *rus4*, or *rus5* mutants displayed no abnormal phenotypes under standard growth conditions. However, homozygous rus6 mutant embryos failed to develop beyond the globular stage, and subsequently their seeds were aborted. *RUS6* expression was detected in many phases of plant development, and was especially strong in flowers. *RUS6* is an essential gene in Arabidopsis embryo development, and is likely to function throughout the plant life cycle. DUF647-containing proteins are ubiquitously present in eukaryotes and our study uncovered an essential role for one of the DUF647-containing proteins in *Arabidopsis* embryo development.

## Method

### Phylogenetic analysis

Sequences were aligned with the MUSCLE program (MUltiple Sequence Comparison by Log-Expectation, https://www.ebi.ac.uk/Tools/msa/muscle/), and subsequently used to construct a neighbor-joining phylogenetic tree with UPGMA clustering method, distance correction and gaps excluded (https://www.ebi.ac.uk/Tools/phylogeny/simple_phylogeny/).

### Plant materials and growth conditions

*Arabidopsis* seeds were surface sterilized and cold treated in darkness at 4 °C for 48 to 72 h, then plated on Murashige and Skoog (MS) medium containing 0.5% D-sucrose, with a full range vitamin supplement minus B6 (except when stated otherwise). *rus6*/ + plants were also germinated directly in potting media with no change in phenotype. Growth conditions for *Arabidopsis* (ecotype Col-0) plants were as described before [7]. Seeds of T-DNA knockout lines for *rus3-1* (SALK_135717C), *rus4-2* (GK_447F02), *rus5-1* (SALK_038772C), *rus6-2* (EMB 1879) were obtained from the ABRC (Arabidopsis Biological Resource Center) at Ohio State University (https://abrc.osu.edu/). The SALK lines were donated by the Salk Institute Genomic Analysis Laboratory. The T-DNA line for *rus6-1* (GK-278G06-015,156) was obtained from the University of Nottingham courtesy of Gabi-Kat (https://www.gabi-kat.de/).

### Seed count

Siliques at mature stages of development (~ 13 mm in length or greater) were dissected under an Olympus SZX12 Stereozoom microscope equipped with a Qimaging micro publisher 5.0 megapixel CCD camera. Images were captured using Qcapture version 2.6 software. We noticed that the less developed siliques contained fewer brown seeds. Of those present, most were similar to the white seeds in appearance, plump with an off-white, or light brown color. In contrast, more developed siliques contained significantly more brown seeds, most of which were of a darker brown shade, and wrinkled. We suspected that a portion of the white aborted seeds took on this brown and wrinkled appearance, which increased in severity as the silique matured. To test this, we counted the number of brown wrinkled seeds present in sequential siliques along an inflorescence stem, thereby enumerating seed color and shape along a spectrum of development. Numbers of brown wrinkled seeds increase with silique development supporting that both white and brown seeds represent the same defect.

### Seed mounting, clearing, and observation

Siliques at mature stages of development (~ 13 mm in length or greater) were dissected under a dissecting microscope. White and green seeds were separated and place directly onto a slide containing a drop of Hoyer’s solution, which was prepared as described in [27], then diluted to one-half concentration. Siliques at immature stages of development (less than ~ 13 mm in length) were dissected directly in a drop of Hoyer’s solution on a slide. All seeds were incubated in solution at RT for 2–16 h until clear. Seeds were observed using a Nikon Eclipse 80i manual upright microscope with Nomarski (DIC) optics. Images were captured using Qcapture version 2.6 software.

### PCR genotyping

DNA was extracted from seedlings grown on MS plates and screened for T-DNA insertions and wild-type alleles. Polymerase chain reaction was used to amplify DNA (see Supplemental Table 1 for primer list) under the following conditions: denaturation 94°c for 5 min, followed by 45 cycles of 60 s at 95 °C, 30 s at 58 °C, 90 s at 72 °C, followed by 10 min at 72 °C. For the RUS6 GK278G06 line, primers were designed to amplify the T-DNA left border and it’s genomic flanking sequence: TJLB 155 and RUS6-1936-F. Primers for the wild-type allele were designed to span both sides of the large (approx. 5,800 bp) T-DNA insertion, permitting amplification of the wild-type allele only: RUS6-1936-F, and RUS6-cds2-R. For the EMB 1879 line, primers were designed to amplify the T-DNA left border and it’s genomic flanking sequence: GARLIC-LB3, and RUS6 RTR. Primers for the wild-type allele were designed to amplify a fragment beginning in the promoter region, and extending into exon 1: RUS6-PROMOTER3-F, and RUS6-R1. Note that exon 1 is part of the proposed deletion in the EMB 1879 line, preventing RUS6-R1 from annealing to the mutant allele. 1% EtBr gels were imaged using the Azure c200 gel imaging workstation.

### Construction of transgenic plant lines

The promoter region of RUS6 was amplified from genomic DNA with primers RUS6-P-Kpn1-Sal1-F and RUS6 P-BamH1 (see Supplemental Table 1 for primer list) and inserted into pCR8. The RUS6 promoter insert was digested from pCR8 at Sal1 and BamH1 and inserted into a modified pBI101 with a GUS reporter completing the construct. For the GFP construct, we amplified RUS6 cDNA from an existing construct using primers AT5G49820-Kpn1-F and AT5G49820-BamH1-R (see Supplemental Table 1 for primer list). This fragment was inserted into pBluescript at Kpn1 and BamH1. We subsequently inserted the RUS6 promoter insert from pCR8 at Sal1 and Kpn1. Finally, we digested the entire RUS6 promoter and cDNA insert at Kpn1 and BamH1 and ligated into a modified pZP222-GFP vector.

### Transformation and complementation

Agrobacterium tumefaciens (GV3101) transformed with the desired constructs was grown for 24 h in 50 mL of LB broth (with antibiotics), after inoculation with a 5 ml starter. Cells were spun-down and resuspended in an equal volume of ddH20 with 0.2% Vac-in-stuff (Silwet L-77). This solution was used to spray Arabidopsis flowers as the avenue of transformation. Transformed plants were incubated under clear plastic at RT in the dark for 24 h then returned to the growth chamber. This method was repeated one week later for a total of two transformation events. *rus6*/ + GK278G06 plants were complemented by the chimeric AT5G498920 (RUS6)-GFP gene driven by a RUS6 promoter in the construct pZP222-GFP. Seeds were harvested and plated on Sul + , Gen + MS media for antibiotic selection. Two T-1 Transformants and multiple T-2 (complemented) progeny were recovered and confirmed by PCR analysis. WT plants were transformed with the modified pBI101 GUS reporter construct driven by a RUS6 promoter using the same methods above. T-1 seeds were harvested and plated on Kan + MS media for antibiotic selection. Twelve T-1 Transformants and multiple T-2 progeny were recovered and confirmed by PCR analysis.

### GUS staining

All plant tissues were vacuum infiltrated for thirty minutes with GUS staining solution, (1 mg 5-bromo-4-chloro-3-indolyl β-D-Glucuronide (X-Gluc) dissolved in 0.1 mL methanol, 1 mL 2 × buffer (20 μL 0.1 M potassium ferrocyanide, 20 μL 0.1 M potassium ferricyanide), 10 μL 10% (w/v) solution of Triton X—100, 0.85 mL water), then incubated overnight at 37 °C. Samples were cleared using 70% ethanol. All samples were observed using an Olympus SZX12 Stereozoom microscope equipped with a Qimaging micro publisher 5.0 megapixel CCD camera. Images were captured using Qcapture version 2.6 software.

### RUS6 subcellular location RUS6-GFP

RUS6-GFP complemented and wild-type control seeds were dissected and mounted in a 6% glycerol solution, embryos were extruded by tapping the slide as described by [27]. Embryos were observed using a Zeiss LSM 710 Confocal Laser Scanning Microscope. GFP fluorescence was excited by a blue argon laser (488-nm blue excitation) and detected at 515- to 530-nm wavelengths. Images were processed using Fiji ImageJ [28].

## Supplementary Information

**Additional file 1.**

## Data Availability

Linked genotype and phenotype data used and/or analyzed during the current study are deposited at TAIR (The Arabidopsis Information Resource—arabidopsis.org). The linked genotype and phenotype for the four genes (RUS3, RUS4, RUS5, RUS6) can be found at these links: RUS3 https://www.arabidopsis.org/servlets/TairObject?id=28854&type=locus RUS4 https://www.arabidopsis.org/servlets/TairObject?id=32619&type=locus RUS5 https://www.arabidopsis.org/servlets/TairObject?id=131341&type=locus RUS6 https://www.arabidopsis.org/servlets/TairObject?id=132107&type=locus

## Abbreviations

DUF: Domain of unknown function
EMB: Embryo-defective
GFP: Green fluorescence protein
GUS: β-Glucuronidase
PLP: Pyridoxal 5-phosphate
MS medium: Murashige and Skoog medium
RUS: Root UV-B sensitive
Sul: Sulfadiazine
WXR: Weak Auxin response
X-Gluc: 5-Bromo-4-chloro-3-indolyl β-D-Glucuronide
