# Supplementary material for: RUS6, a DUF647-containing protein, is essential for early embryonic development in Arabidopsis thaliana
Source: BMC Plant Biol. 2021 May 25;21:232. doi: 10.1186/s12870-021-03011-8 (PMC8146622; doi:10.1186/s12870-021-03011-8)
Supplement: Supplementary file 1 — Additional file 1. [file 12870_2021_3011_MOESM1_ESM.pdf]

Perry et al., RUS6, a DUF647-containing protein, is essential for early embryonic development in *Arabidopsis thaliana*

Supplementary Information

(Figs with Fig legends, and Table)

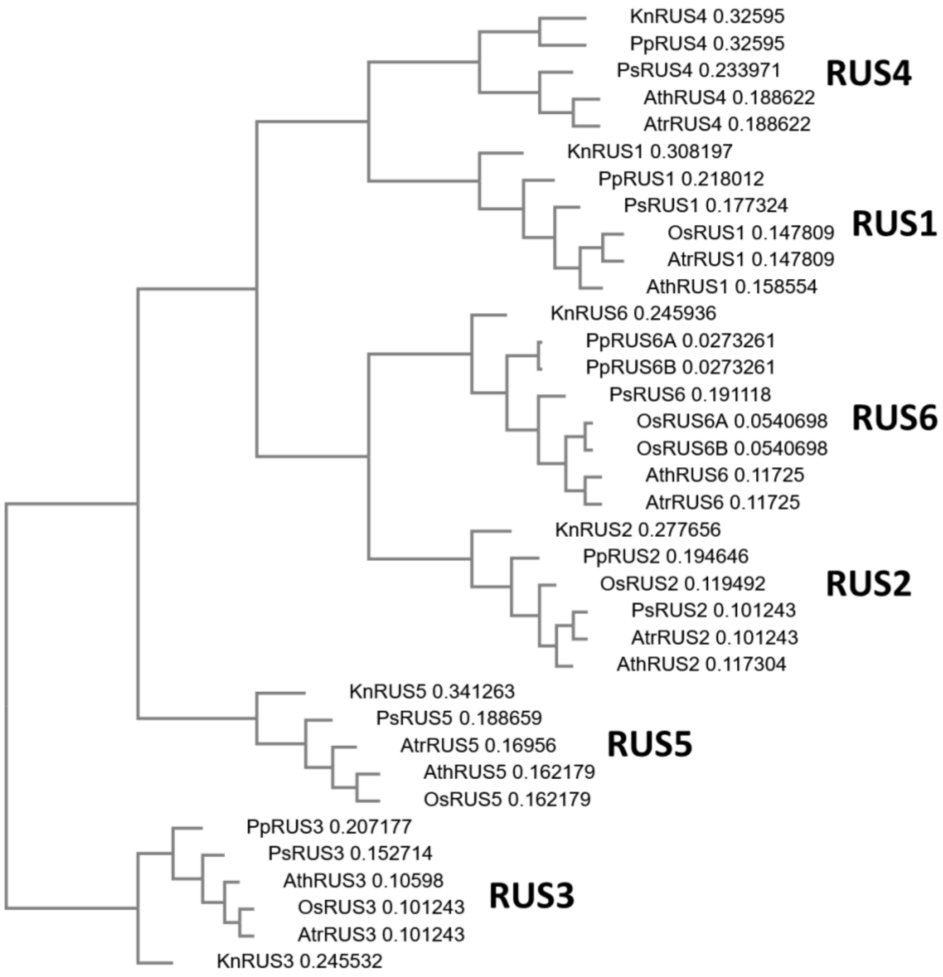

Fig. S1. Phylogeny of the RUS family. Phylogenetic analysis of full-length RUS proteins from various species shows that all RUS proteins cluster into one of six groups, numbered for the *Arabidopsis* proteins. Ath = *Arabidopsis thaliana*, Atr = *Amborella trichopoda*, Os = *Oryza sativa*, Ps = *Pinus sylvestris*, Pp = *Physcomitrella patens*, Kn = *Klebsormidium nitens.*

A

5'…TGGGGCAAACCAGCGTGGACCGCT
TGCTGCAACTCTCTCAGGGCCAGGCG
GTGAAGGGCAATCAGCTGTTGCCCGTC
TCACTGGTGAAAAGAAAAACCACCCCA
GTACATTAAAAACGTCCGCAATGTGTTA
TTAAGTTGTCTAAGCGTCAATTTGTTTA
CACCACAATATATCCTGCCACCAGCCA
GCCAACAGCTCCCCGACCGGCAGCTC
GGCACAAAATCACCACTCGATACAGGC
AGCCCATCAGTCATTTAGTGCGTATCAT
TTCTTGCATCAAACATTGAAGTTAAGAC
TTCGGTTTGGATTGATACATCGGTTTTT
AACTTTTTTCAATCTTGGCTATTAGCTG
GTGTTGCAAGTGGAGCAACCAGAGCA
GCTCTAACACAGCACTTTGCTCTTCAA
GACAATGCAGCGGATATATCTGCTAAG
GTACTCTTTACTTCTTGTCACTGTGAAT
GATATAGAAACTATAAATGTGAACTTTG
AGTAATTCTTTG... 3'

B

5'…aagatttccgaattagaataatttgttt
attgctttcgcctataaatacgacggatcgt
aatttgtcgttttatcaaaatgtactttcatttta
taataacgctgcggacatctacatttttgaat
tgaaaaaaaattggtaattactctttctttttct
ccatattgaccatcatactcattgctgatcc
atgtagatt*ATTGAAGCCATGTA
GCCATATTAACTATCAGTGTA
CTTGGAAGGTTAAGTAGATG
TATATACACT*GCTAGTCTTG
CTTCTGCTTTTGACACTAATT
TGAAGGTATTTTACTTCACTA
AATTATAGTTCTTGAAACTTT
GGAGATTTTTTGTTACTCATA
TACTCTTAGGATTTTGCTAAA
TCAAGAGAGTTAGGTTCTCG
ACGTCTGTACTCTTCAGTCTA
AGCATTGGAGTTGAGTTAAT
GACTCCAGTATTCCCTCAGTA
CTT...3'

C

5'...CCATTTTGTTTTGCCTTGCCTTTAA
TTCATGAATGTAAAACAAATAATGCAG
GTGAATGCCACAAGCAGAGACGTGTT
GAGATGCCTTTGGCAAGCATATTGGTT
AGAGGAGAACATGGAAGAAAGCTTTA
AAGACAAGGATAGTGTGTTCCACTGG
CTGAAGCAAAGCTTGTCGGAGATGGA
CAACAAGTTTGATGATTTCTTGTTCAA
ATTGGATACTGCTGGATGGAATCTAC
GTGAATCTAACTTGAGCTTAGACAACT
TAATAACACATTGCGGACGTTTTTAAT
GTACTGGGGTGGTTTTTCTTTTCACCA
GTGAGACGGGCAACAGCTGATTGCCC
TTCACCGCCTGGCCCTGAGAGAGTTG
CAGCAAGCGGTCCACGCTGGTTTGCC
CCAGCAGGCGAAAATCCTGTTTGATG
GTGGTTCCGAAATCGGCAAAATCCCT
TATAAATCAAAAGAATAGCCCGAGATA
GGGTTGAGTGTTGTTCCAGTTTGGAA
CAAGAGTCCACTATT...3'

D

3'...ACTTTCCATCTATCTGCAGGGCGGGTTCCCTCATTGCAGGAA
GGTAATATCCAAGAAAAGATATTCACTTTTCCATGGGTGGATGAT
CGGCCAGTGATGCTTGGTATGTGTTATCAAGCATTCCATATAAAT
TATATTCCTTTTTCATTACCCTTACTGATTAGGTTTTTCATACAATT
TGACAGGAGCCAGATTTAAGGATGCATTCCAAGACCCCAGCACG
TATATGGCTGTAAAGCCTTTTTTCGATGTATGTGTTTTTTCCCTTA
ATATCAAGCCTTGACCGCTTTATCTTGATACTGTAATTGACTTTAT
TCTCTTTACTAATGGCAGAAAGAAAGA*GTAATGAGTAATGAGTA
AGGACAT*GGCAGGATATATTCAATTGTAAATGGCTTCATGTCCG
GGAAATCTACATGGATCAGCAATGAGTATGATGGTCAATATGGA
GAAAAAGAAAGAGTAATTACCAATTTTTTTTCAATTCAAAAATGTA
GATGTCCGCACGGTTATTAT… 5'

E

3'...TAGCATCTGAATTTCATAACCAATCTCGATACACCAAATC
GAATTCAATTCGGCGTTAATTCAGTACATTAAAAACGTCCGCA
ATGTGTTATTAAGTTGTCTAAGCGTCAATTTGGGTGTTTTGTT
GGTTTAATCTGTCAAGCGGAATTAAATTTGAACTAAAGGTGG
CTACATTGCTAGTTTGCTACTTGTTGCTTAAGGGTCGAAATAG
ATTCTGTTTGATCTCTTTTTGAGCTCTAAAATTTTCATTTGTTT
TTATTATTTTCTATGTGAAGATGGGAACTGGGTTTAGCATCCT
GATATCAAAAAGAAATCCTTCGTTGGTCACAACATTTGGTCTT
CTATCATGTGGCTATCTCATGAGCTCGTACCAAGAGGTAGAA
TCCAGCTCTGTTTCAAGTATATCTCTTTTTATGTACTTGGGGT
TCTCTTGATTTATTGCTCAAAAAACGTGGCTGTGTCCTTTACA
GGTTAGATCTGTA... 5'

Fig. S2. Confirmation of T-DNA flanking sequences in knockout mutants. A. T-DNA flanking sequences in *RUS3*. Partial sequences in transgenic line, *SALK_135717C (*pROK2 system) *(rus3-1*) is shown. **B.** T-DNA flanking sequences in *RUS4*. Partial sequences in transgenic line, *GK-447F02-024530 (*pAC161 system) (*rus4-2*) is shown. **C.** T-DNA flanking sequences in *RUS5*. Partial sequences in transgenic line, *SALK_038772C/*pROK2 *(*pROK2 system) *(rus5-1*) is shown. **D.** T-DNA flanking sequences in *rus6-1*. Partial sequences in transgenic line, *GK278G06/*pAC161 (*rus6-1*) is shown. **E.** T-DNA flanking sequences in *rus6-2*. Partial sequences in transgenic line, *EMB 1879/*pCSA104 (*rus6-2*) is shown. The underlined portion represents *RUS* genomic DNA (highlighted in blue). The not underlined portion represents the LB of the T-DNA insertion vector (highlighted in red). The sequence between the stars * is assumed to be related to the T-DNA insertion (highlighted in orange), but does not align with the T-DNA or *RUS* genomic DNA.

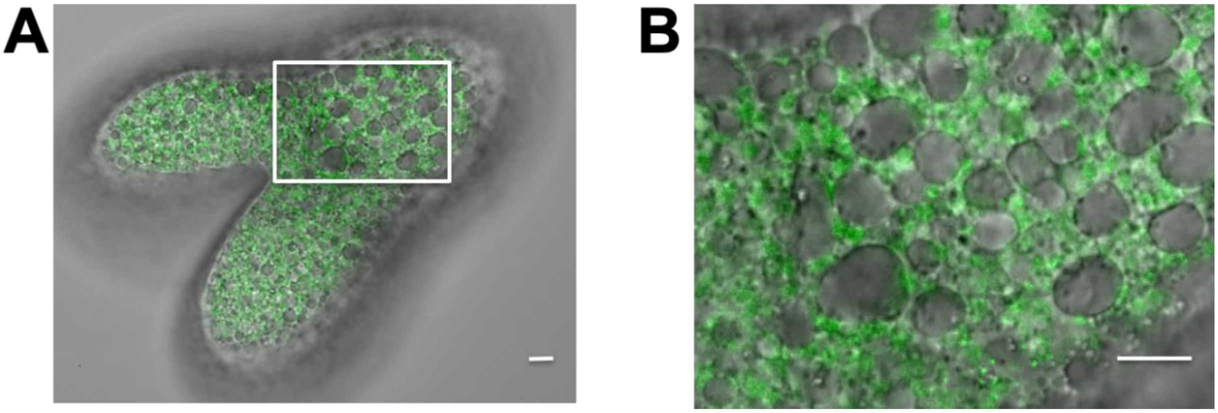

Fig. S3. Expression of RUS6-GFP in cells of developing embryo. Embryos from a RUS6::RUS6-GFP transgenic line (in homozygous *rus6-1* background) were dissected and examined under a Confocal microscope. **A.** A whole embryo view of RUS6-GFP expression. **B.** Closeup view of the inset in (A) 40x (Bar = 10 microns).

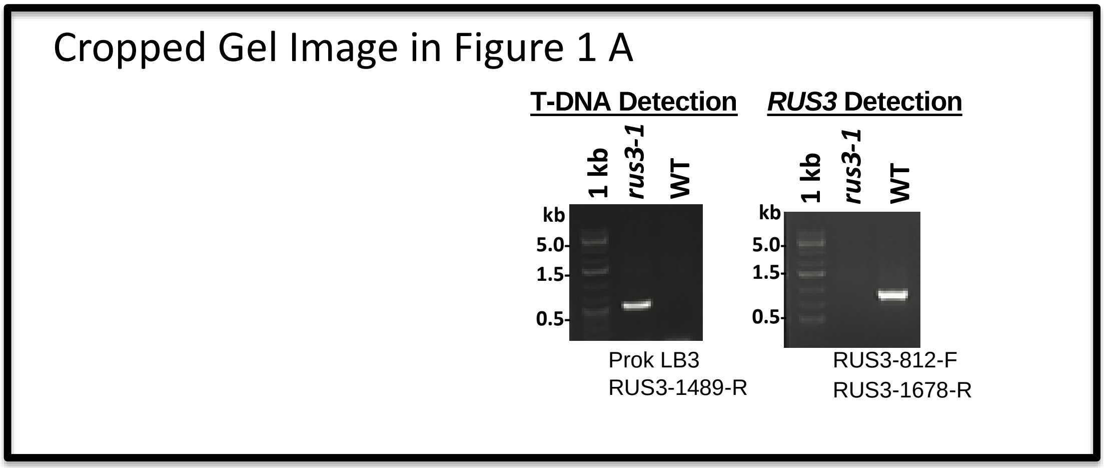

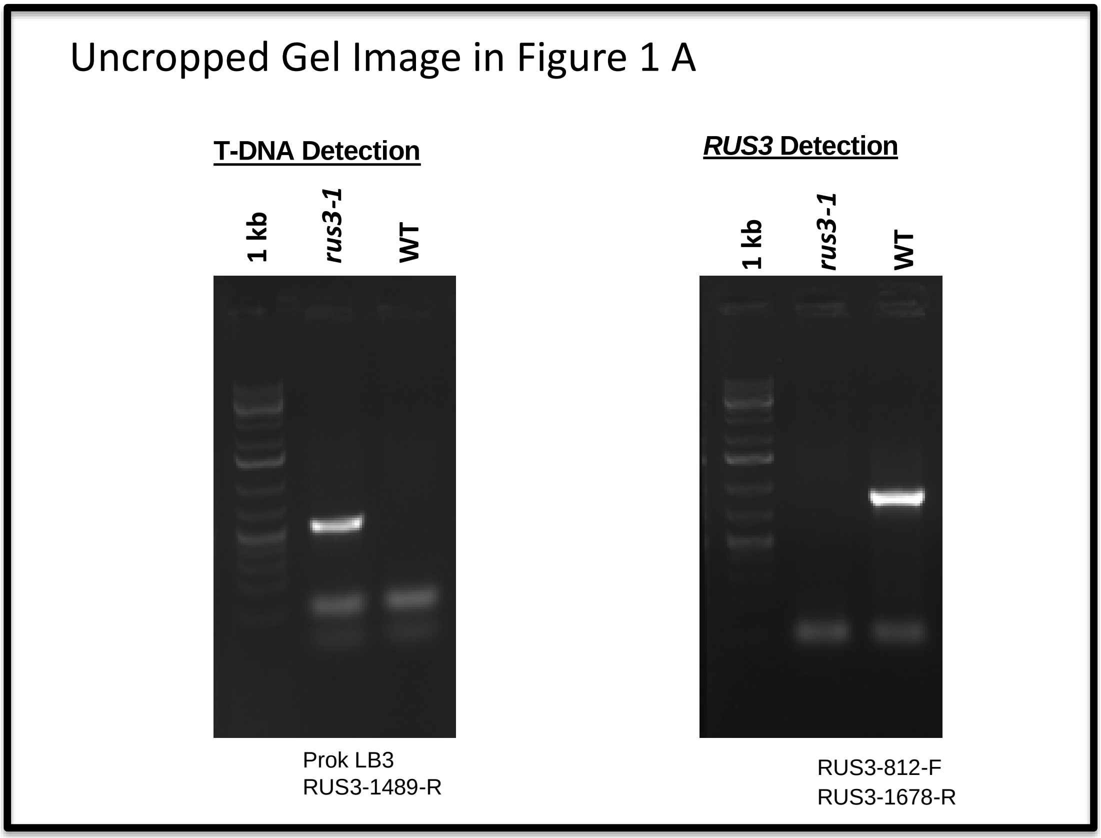

Fig S4. Uncropped gel images for Figure 1A. Top panel, cropped gel image shown in Figure 1 A. Bottom panel, uncropped original gel images for Figure 1A.

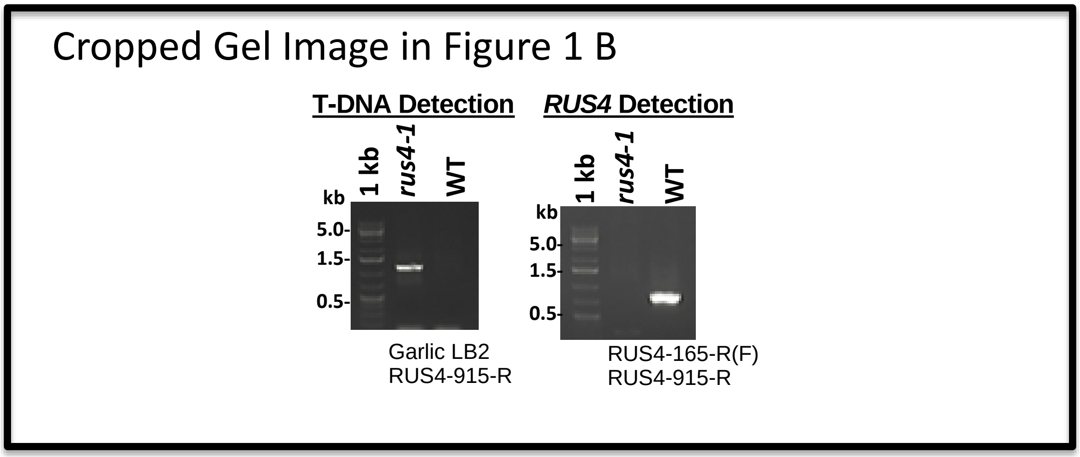

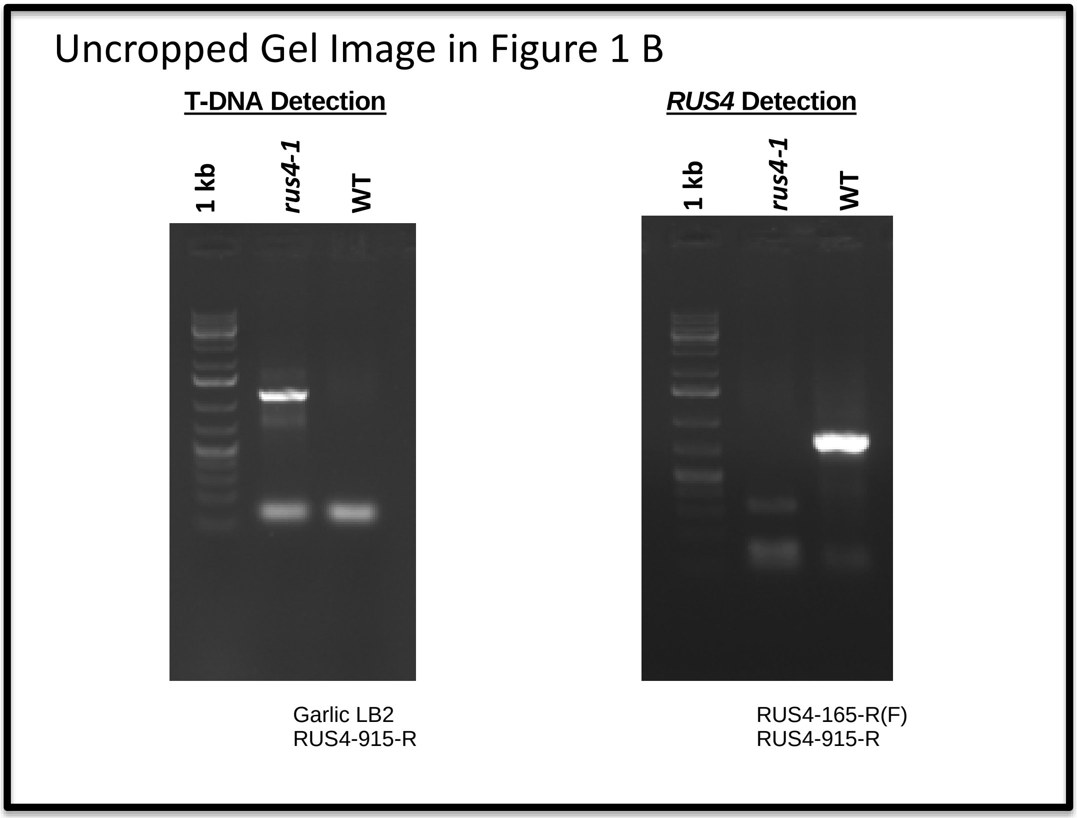

Fig S5. Uncropped gel images for Figure 1B. Top panel, cropped gel image shown in Figure 1 B. Bottom panel, uncropped original gel images for Figure 1B.

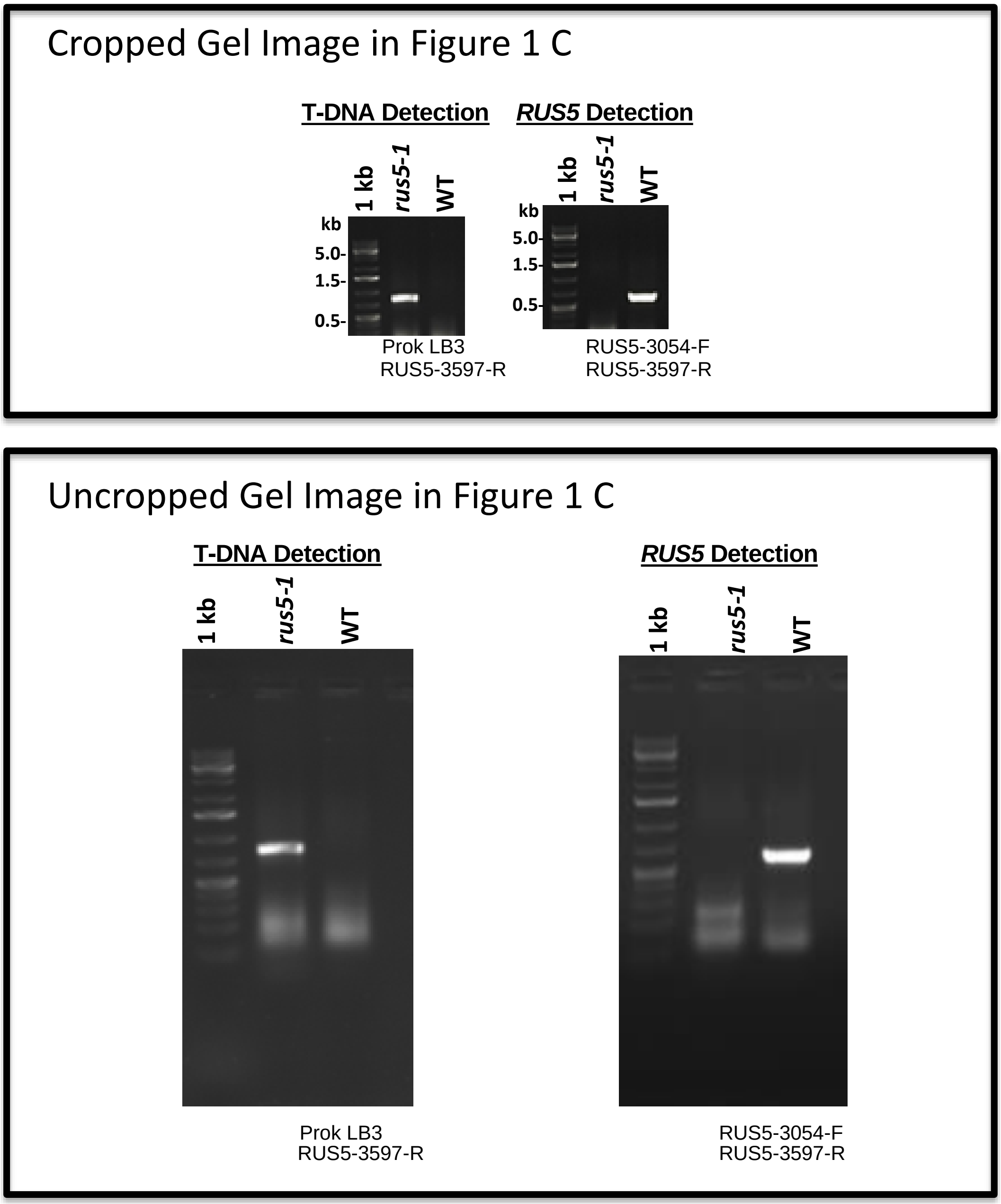

Fig S6. Uncropped gel images for Figure 1C. Top panel, cropped gel image shown in Figure 1 C. Bottom panel, uncropped original gel images for Figure 1C.

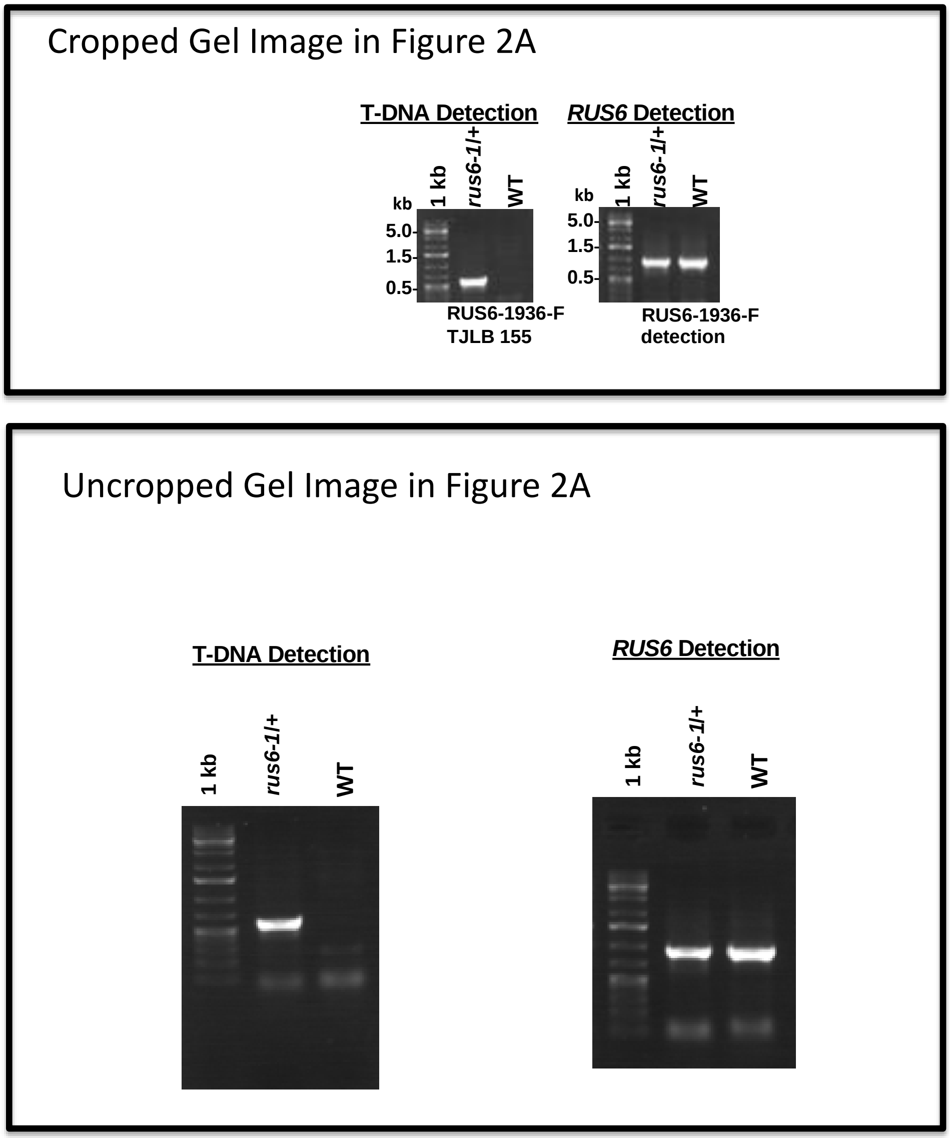

Fig S7. Uncropped gel images for Figure 2A. Top panel, cropped gel image shown in Figure 2A. Bottom panel, uncropped original gel images for Figure 2A.

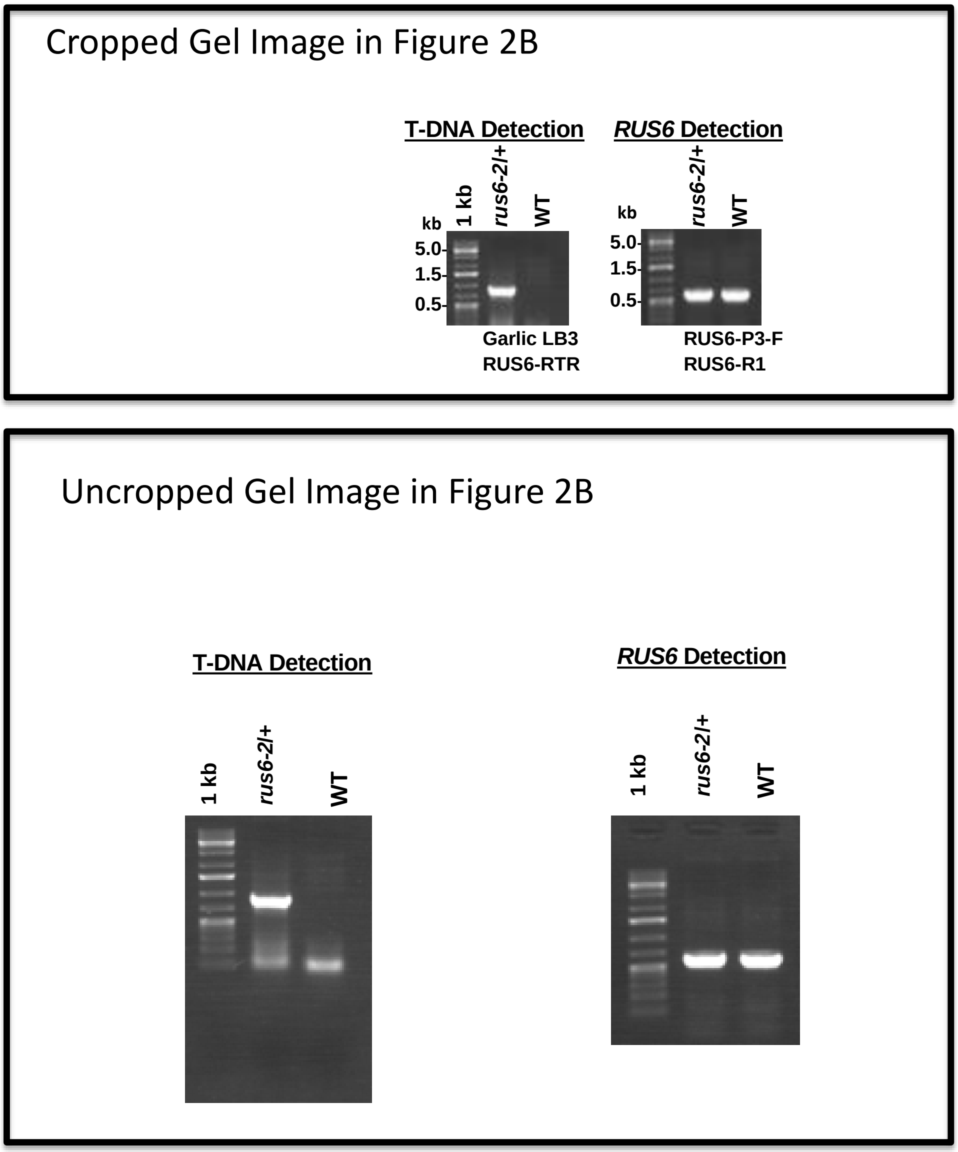

Fig S8. Uncropped gel images for Figure 2B. Top panel, cropped gel image shown in Figure 2B. Bottom panel, uncropped original gel images for Figure 2B.

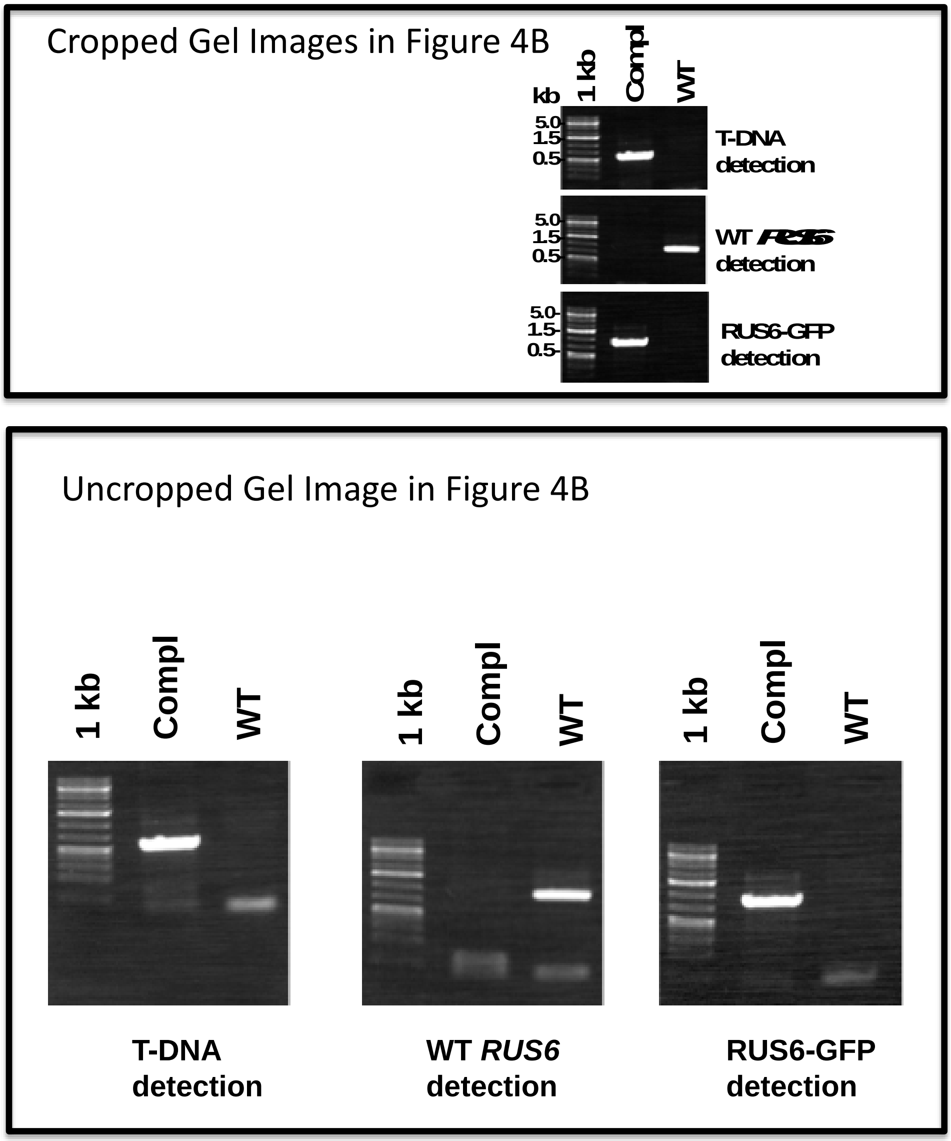

Fig S9. Uncropped gel images for Figure 4B. Top panel, cropped gel image shown in Figure 4B. Bottom panel, uncropped original gel images for Figure 4B.

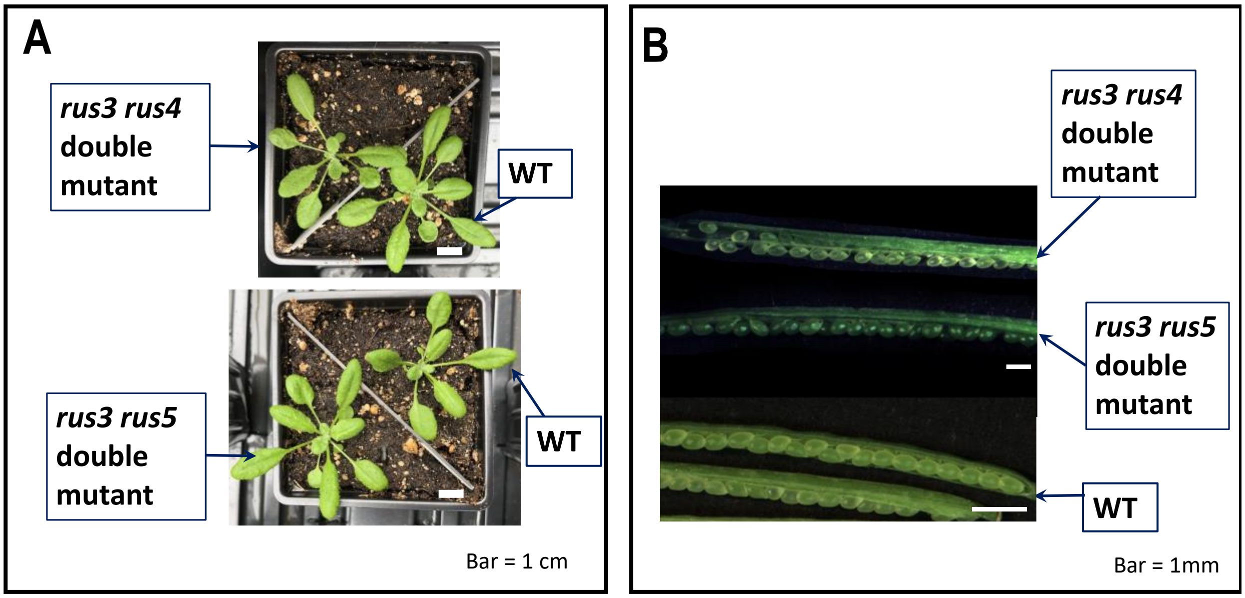

Fig. S10. Double mutants (*rus3 rus4* and *rus3 rus5*) are morphologically indistinguishable to WT. A. Three-week-old double mutant seedlings were grown side-by-side with WT (as indicated). Top: *rus3 rus4* ; Bottom: *rus3 rus5*. Bar = 1 cm. **B.** Dissected siliques from *rus3 rus4* **,** *rus3 rus5* and WT (as indicated). Bar = 1 mm.

S1 Table. List of primers Used in this study. Primers used for PCR markers to detect T-DNA insertions or the WT gene fragments are listed here. Primers used to make various constructs are also listed.

PRIMER NAME	PRIMER SEQUENCE	SEED LINE/CONSTRUCT	Pair for T-DNA or WT?	Approx expected size in bp
**RUS3**				
PROKLB3	CCTGATAGACGGTTTTTCGC	SALK_135717C	T-DNA	484
RUS3-1489-R	GTGCTGTGTTAGAGCTGCTC			
RUS3 -812-F	CCGCAACTGTTATAGGTGCTAC		WT	867
RUS3-1678-R	GAGCTAGCAACATTCCCAATGAC			
**RUS4**				
Garlic LB2	GCTTCCTATTATATCTTCCCAAATTACCAATACA	GK-447F02-024530	T-DNA	1,058
RUS4-915-R	AAACGTTGCTAAGTAGCACGACAG			
RUS4-165-R (actually Forward)	AAACCGTAGTACCCAGTAACGTAG	GK-447F02-024530	WT	759
RUS4-915-R	AAACGTTGCTAAGTAGCACGACAG			
**RUS5**				
PROKLB3	CCTGATAGACGGTTTTTCGC	SALK_038772C	T-DNA	762
RUS5-3054-F	GTTTATTCACCTTGCTGGAGAG			
RUS5-3054-F	GTTTATTCACCTTGCTGGAGAG	SALK_038772C	WT	543
RUS5-3597-R	CTGTTCAAGAGATTGCGTGATG			
**RUS6**				
TJLB 155	ATAACGCTGCGGACATCTAC	GK-278G06	T-DNA	592
RUS6-1936-F	CTAGCATGCACTTGAGTGTGC			
RUS6-1936-F	CTAGCATGCACTTGAGTGTGC	GK-278G06	WT	932
RUS6-cds2-R	CTCCCCCTCCTTCACATGACATAAC			
Garlic LB3	TAGCATCTGAATTTCATAACCAATCTCGATACAC	EMB 1879	T-DNA	874
RUS6-RTR	GCCATATACGTGCTGGGGT			
RUS6-promoter3-F	ATCAGGGTGCAAAACACAACA	EMB 1879	WT	569
RUS6-R1	CATCGCTCTCAGCGACATAC			
**Transgenic plant constructs**				
RUS6-P-KPN1-Sal1-F	GTCGACGGTACCGAATCATGTAGTTCATAGCGT	pBI101-RUS6P-GUS and		
RUS6-P-BamH1-R	GGATCCTGGCATTGTTGTTGTTGTAAATCT	pZP222-RUS6P-RUS6CDS-GFP		
ATG49820-Kpn1-F	ACAGGTACCATGCCAAGCGTCAAGCTCACA	pZP222-RUS6P-RUS6CDS-GFP		
AT5G49820-BamH1-R	GCGGGATCCACTCCCCCTCCTTCACATGAC			
